# Supplementary material for: A unique Co@CoO catalyst for hydrogenolysis of biomass-derived 5-hydroxymethylfurfural to 2,5-dimethylfuran
Source: Nat Commun. 2022 Jun 27;13:3657. doi: 10.1038/s41467-022-31362-9 (PMC9237033; doi:10.1038/s41467-022-31362-9)
Supplement: Supplementary file 1 — Supplementary Information [file 41467_2022_31362_MOESM1_ESM.pdf]

Supplementary Information

A Unique Co@CoO Catalyst for Hydrogenolysis of Biomass-derived 5-Hydroxymethylfurfural to 2,5-Dimethylfuran

Shuang Xiang[1,δ], Lin Dong[1,δ], Zhi-Qiang Wang[1,δ], Xue Han[2], Luke L. Daemen[3], Jiong Li[4], Yongqiang Cheng[3], Yong Guo[1], Xiaohui Liu[1], Yongfeng Hu[5], Anibal J. Ramirez-Cuesta[3], Sihai Yang[2]*, Xue-Qing Gong[1]* and Yanqin Wang[1]*

[1] Key Laboratory for Advanced Materials and Joint International Research Laboratory of Precision Chemistry and Molecular Engineering, Feringa Nobel Prize Scientist Joint Research Center, Research Institute of Industrial Catalysis, School of Chemistry and Molecular Engineering, East China University of Science and Technology, Shanghai, 200237, China.

[2] Department of Chemistry, University of Manchester, Manchester, M13 9PL (UK)

[3] Neutron Scattering Division, Neutron Sciences Directorate, Oak Ridge National Laboratory, Oak Ridge, TN 37831 (USA)

[4] Shanghai Synchrotron Radiation Facility, Shanghai Advanced Research Institute, Chinese Academy of Sciences, Shanghai 201210, China

[5] Sinopec Shanghai Research Institute of Petrochemical Technology, Shanghai 201208, China

[δ] These authors contributed equally

Supplementary Information

Table of Contents

Supplementary Methods 3

Supplementary Figures 5

Supplementary Figure 1 5

Supplementary Figure 2 6

Supplementary Figure 3 7

Supplementary Figure 4 8

Supplementary Figure 5 9

Supplementary Figure 6 10

Supplementary Figure 7 11

Supplementary Figure 8 12

Supplementary Figure 9 13

Supplementary Figure 10 14

Supplementary Figure 11 15

Supplementary Figure 12 16

Supplementary Figure 13 17

Supplementary Figure 14 18

Supplementary Figure 15 19

Supplementary Figure 16 20

Supplementary Figure 17 21

Supplementary Figure 18 22

Supplementary Figure 19 23

Supplementary Figure 20 24

Supplementary Tables 25

Supplementary Table 1 25

Supplementary Table 2 26

Supplementary Table 3 27

Supplementary Table 4 28

Supplementary Table 5 29

Supplementary References 30

Supplementary Methods

Materials

5-Hydroxymethyltetrahydrofurfural (HMF, 99.0%) was purchased from Shanghai De-Mo Pharmaceutical Science and Technology Co., Ltd. 2,5-Furandimethanol (BHMF, 98%+), Dodecane (99.0%) and Cobalt(II) nitrate hexahydrate (99.0%) were purchased from Shanghai Titan Scientific Co., Ltd. 5-Methyl-2-furanmethanol (HMMF, 97.0%) and 1,4-Dioxane were purchased from Shanghai Macklin Biochemical Co., Ltd. 2,5-Dimethylfuran (99.0%) was purchased from Aladdin Reagent Co., Ltd. Tetrahydrofuran (THF, ⩾99.5%), Methyl Alcohol (⩾99.5%) and NH_3CO_3(NH_3⩾40.0%) were purchased from Shanghai Titan chem Co., Ltd. $RuCl_3 \cdot 3H_2O$ and $Pt(NO_3)_2$ were purchased from Heraeus Materials Technology Shanghai Co., Ltd. All chemicals were used as received without further purification.

Catalyst characterization

Powder X-ray diffraction (XRD) patterns were recorded in the θ-2θ mode on a D8 Focus diffractometer (CuKa1 radiation, k = 1.5406 Å), operated at 40 kV and 40 mA within scattering angels of 10–80°.

Transmission electron microscopy (TEM) images of samples were obtained on a JEOL Model 2100F electron microscopy at 200 kV.

X-ray photoelectron spectra (XPS) were recorded on a Thermo Scientific Escalab 250 Xi spectrometer equipped with monochromatic Al Kα radiation, and the results were calibrated by a C 1s peak at 284.6 eV.

Catalyst activity tests in fixed-bed reactor

The hydrogenolysis of HMF was also tested in a fixed-bed reactor system. Prior to the test, 0.3 g of catalyst with 40-60 mesh packed into the middle portion of the stainless-steel tubular reactor (inner diameter of 6 mm, length of 55 cm) was in situ activated at 250 °C for 2 h with H_2 under atmospheric pressure. Then a feed of a 0.03 g/ml HMF in THF was injected into the reactor by an HPLC pump, which resulted in a weight-hourly space velocity (WHSV) of 26.6 h^{-1}. The hydrogenolysis of HMF was conducted at 130 °C and 1MPa H_2 with the H_2 flow rate of 30 mL/min. The liquid phase was separated from gas phase and collected by a gas-liquid separator. The liquid phase analysis was performed with an Agilent 7890A GC-FID instrument equipped with an HP-5 column.

Catalyst activity tests in batch reactor for the hydrogenolysis of the lignin β-O-4 model compound

Hydrogenolysis of the lignin β-O-4 model compound (2-(2-methoxyphenoxy)-1-phenylethanol) was carried out in a Teflon-lined stainless-steel autoclave (50 mL). Typically, 2-(2-methoxyphenoxy)-1-phenylethanol (0.2 g) and the catalyst (0.1 g) were mixed with 1,4-dioxane (5 mL) in the autoclave. Then, the reactor was sealed, purged three times with hydrogen, and charged to 0.5 MPa H_2. Finally, the reactor was heated to 180 °C under magnetic stirring at 600 rpm and kept for a certain reaction time. After reaction, the reactor was quenched in an ice-water bath immediately. The liquid phase was separated from the solid catalyst by centrifugation, and two individual GC/GC-MS systems were used for product analyses. The qualitative analysis of products was carried out on a GC-MS system (Agilent 7890A-5975C), and the quantitative analysis was executed on a GC system (Agilent 7890B) equipped with an HP-5 column and an FID detector.

Supplementary Figures

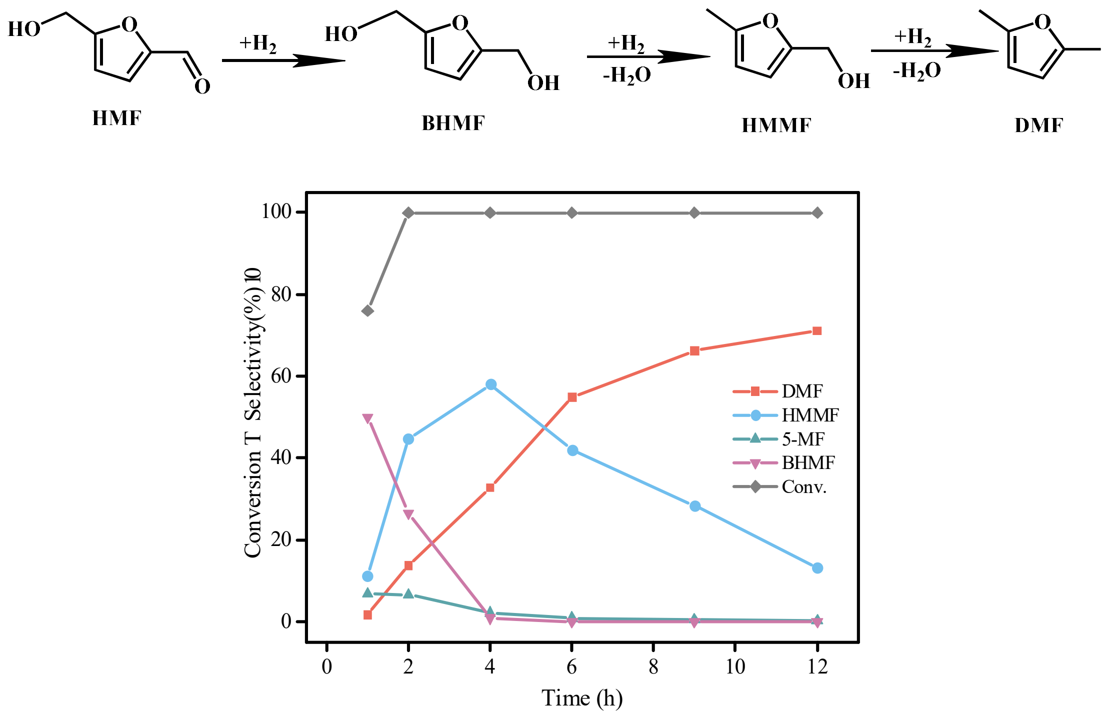

Supplementary Figure 1. Influence of reaction time on hydrogenolysis of HMF over Co_3O_4-250. Reaction condition: HMF, 150 mg; catalyst, 30 mg; THF, 5ml; temperature, 100 °C; H_2, 1.0 MPa.

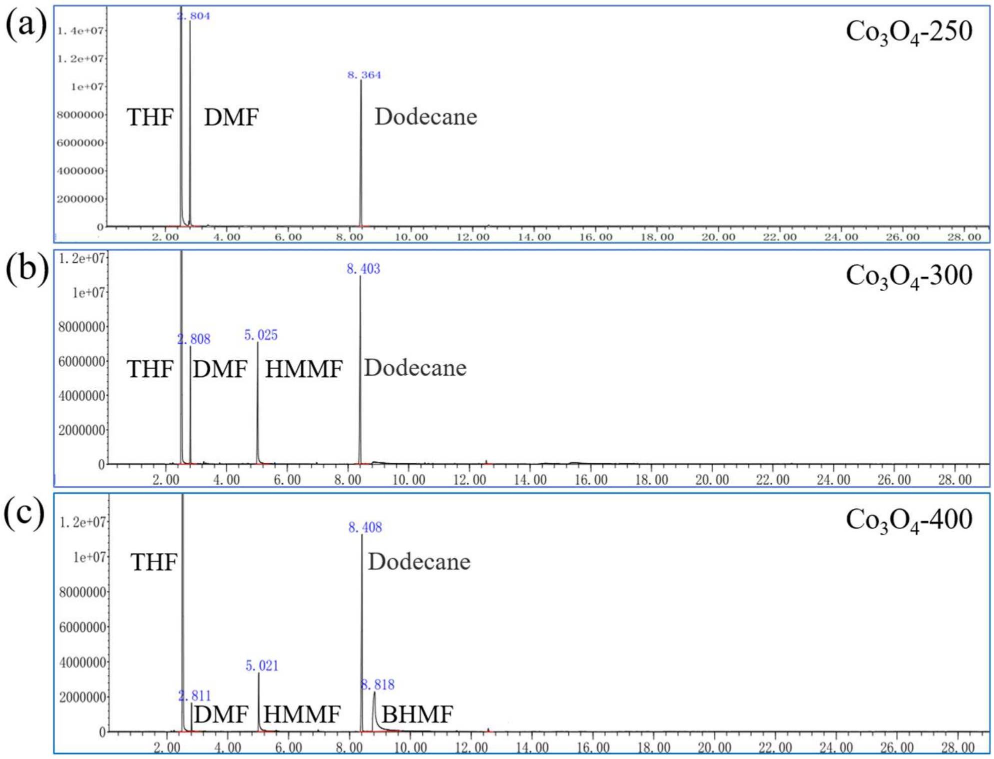

Supplementary Figure 2. GC-MS/FID spectra of the HMF hydrogenolysis reaction over different catalysts. (**a**) Co_3O_4-250, (**b**) Co_3O_4-300 and (**c**) Co_3O_4-400.

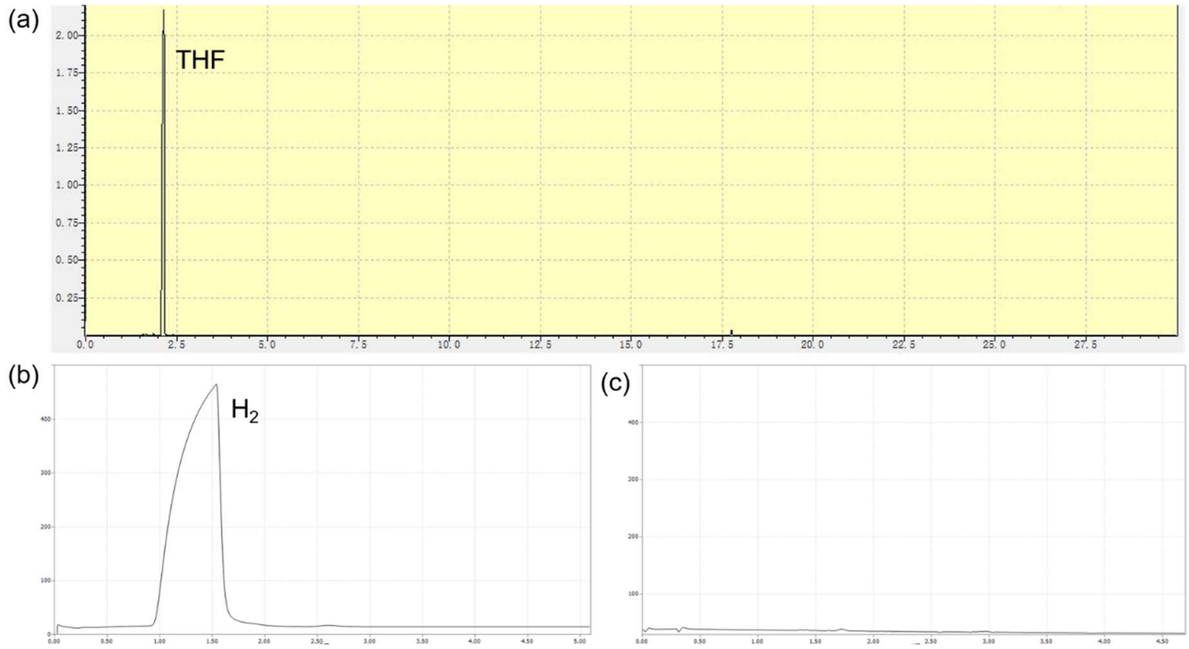

Supplementary Figure 3. Potential products analysis during THF hydrogenolysis. (**a**) GC-MS plot of liquid products, (**b**) GC plot with TCD detector of gas products and (**c**) GC plot with FID detector of gas products. Reaction conditions: Co_3O_4-250: 0.03 g, THF: 5 mL, H_2: 1.0 MPa, 130°C, 2h.

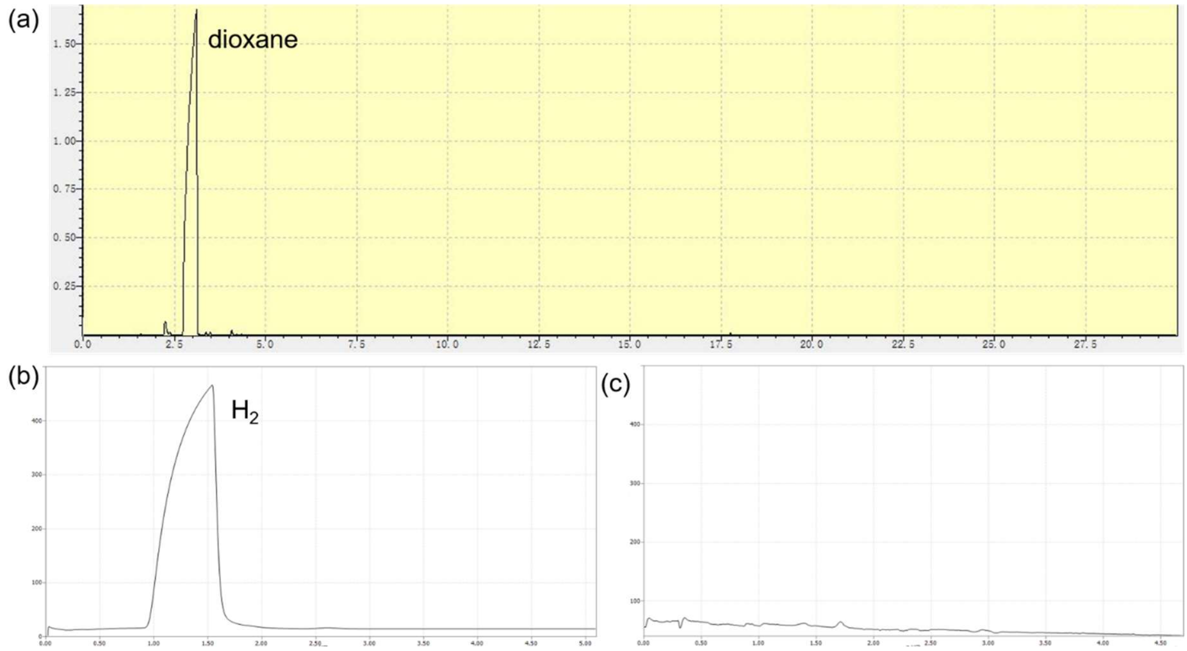

Supplementary Figure 4. Potential products analysis during dioxane hydrogenolysis. (**a**) GC-MS plot of liquid products, (**b**) GC plot with TCD detector of gas products and (**c**) GC plot with FID detector of gas products. Reaction conditions: Co_3O_4-250: 0.03 g, dioxane: 5 mL, H_2: 1.0 MPa, 130 ºC, 2h.

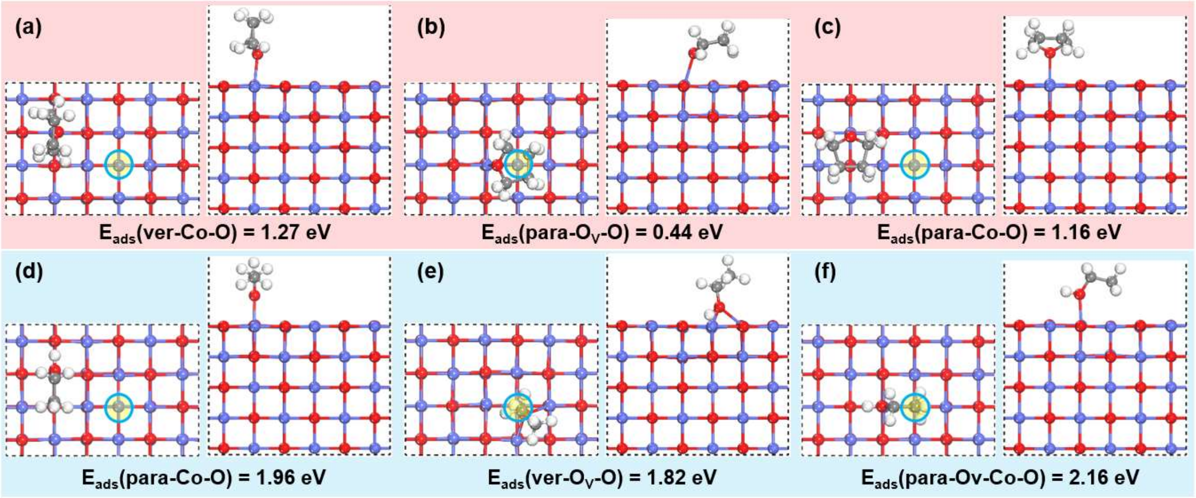

Supplementary Figure 5. Calculated adsorption energies and structures. (**a**)–(**c**) THF (left: top view, right: side view) at the CoO(100)-O_V surface, which are marked with red background; (**d**)–(**f**) ethanol (left: top view, right: side view) at CoO(100)-O_V surface, which are marked with blue background.

The calculated adsorption modes of these solvent molecules (THF and ethanol) on CoO(100)-O_V surface (Supplementary Figure 5) show that: (i) the calculated highest adsorption energies of THF and ethanol on the CoO(100)-O_V surface are 1.27 eV and 2.16 eV, respectively, both of which are below that of HMF (2.22 eV); (ii) as expected, the adsorption energy of ethanol is very close to that of HMF, and the competitive adsorption may lead to the low yield of DMF in alcohol solvents (Supplementary Table 1). Thus, these results indicate that the solvent can influence the catalytic performance, and THF may show the best performance due to its weak adsorption on the CoO(100)-O_V surface.

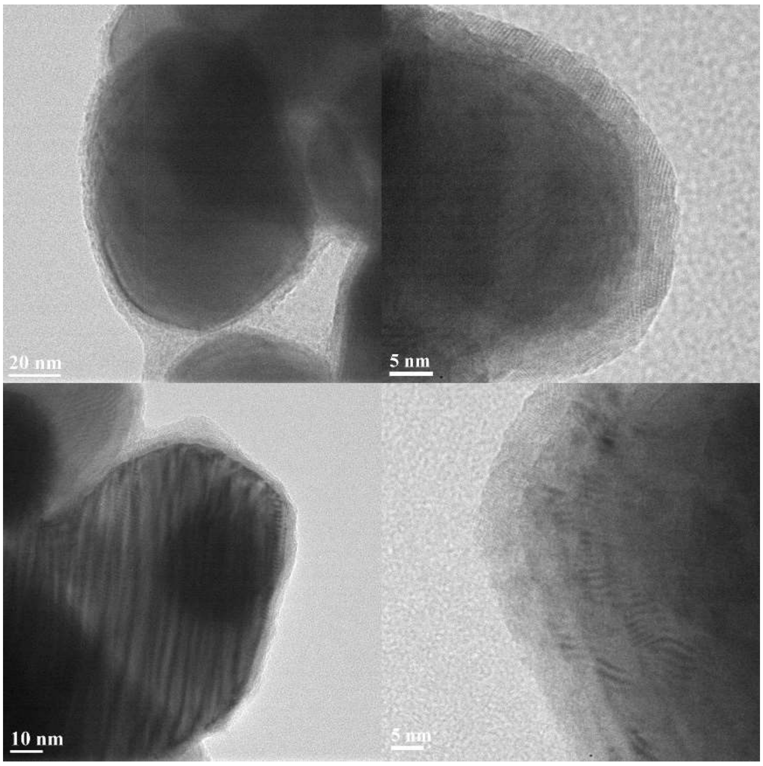

Supplementary Figure 6. Representative HRTEM images of Co_3O_4-250 after fix-bed reaction.

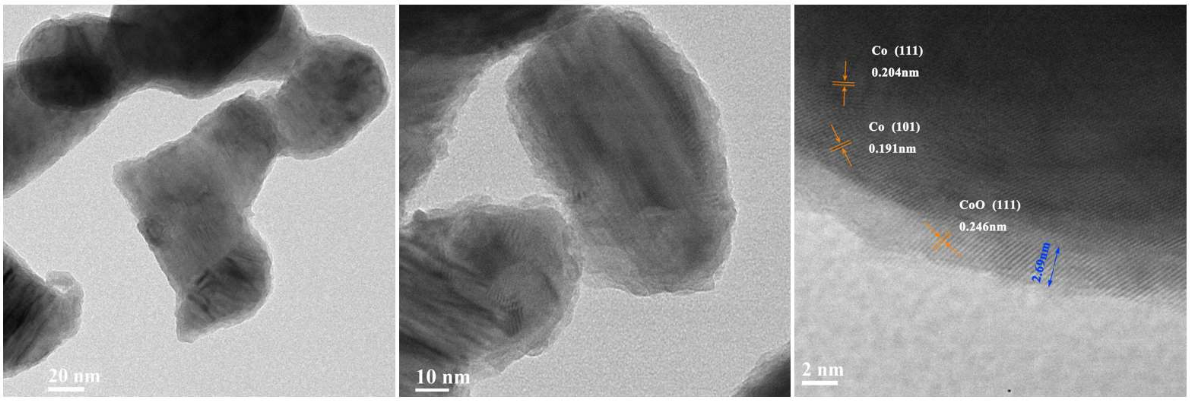

Supplementary Figure 7. Representative HRTEM images of Co_3O_4-250.

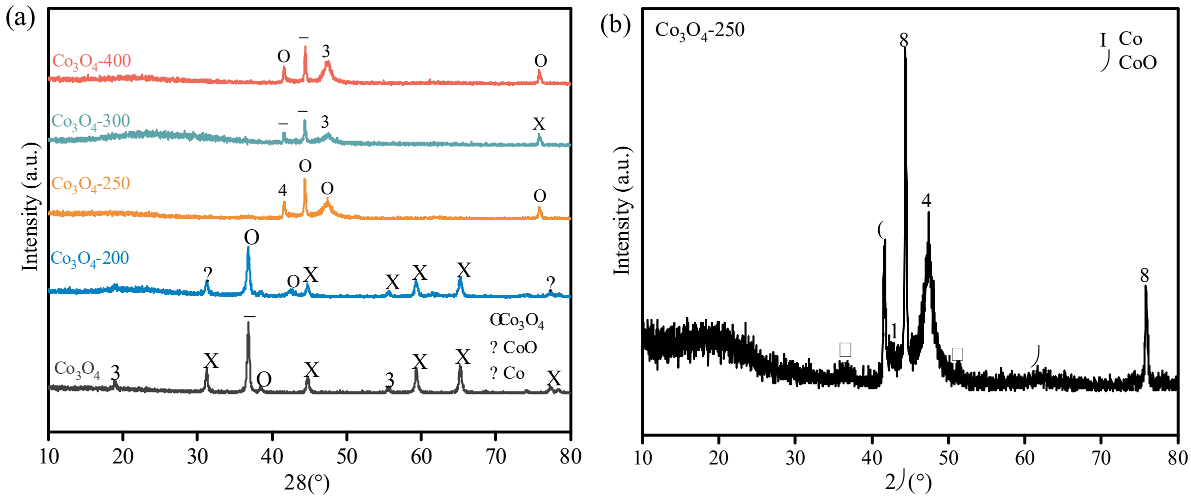

Supplementary Figure 8. XRD patterns of various catalysts. (**a**) Co_3O_4, Co_3O_4-200, Co_3O_4-250 and Co_3O_4-300; (**b**) Enlarged pattern of Co_3O_4-250.

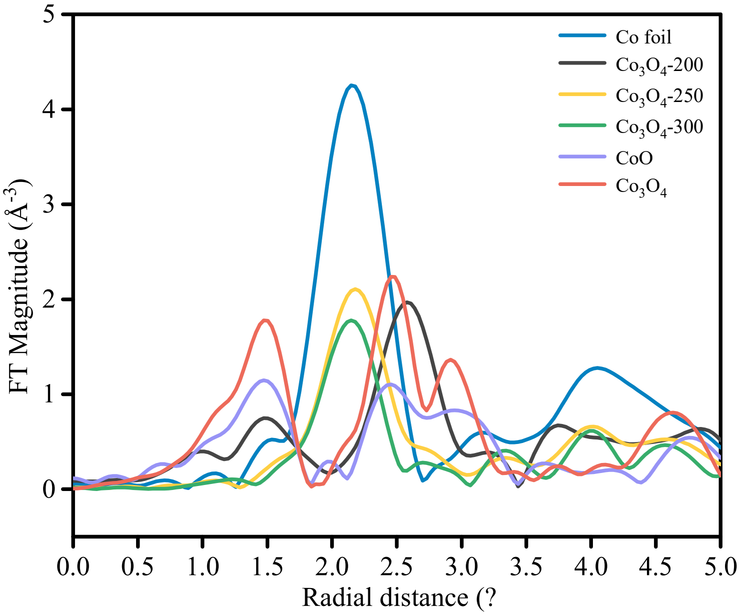

Supplementary Figure 9. EXAFS spectra in R space of Co_3O_4-200, Co_3O_4-250, Co_3O_4-300, Co foil, CoO and Co_3O_4.

The local environment of Co in Co_3O_4-250 and Co_3O_4-300 is similar to that of Co foil with a dominant Co-Co feature at 2.18 Å, but the amplitude of this feature is much lower for the Co_3O_4-250 and Co_3O_4-300, indicating the metallic Co in these two catalysts are not as well ordered as metallic Co. Additionally, a weak signal at 2.70 Å is observed over Co_3O_4-250, similar to the Co-O bond in the CoO standard, confirming that the sample is not reduced completely. These results suggest that the metallic Co and CoO species co-exist in Co_3O_4-250, which are in agreement with the HRTEM results.

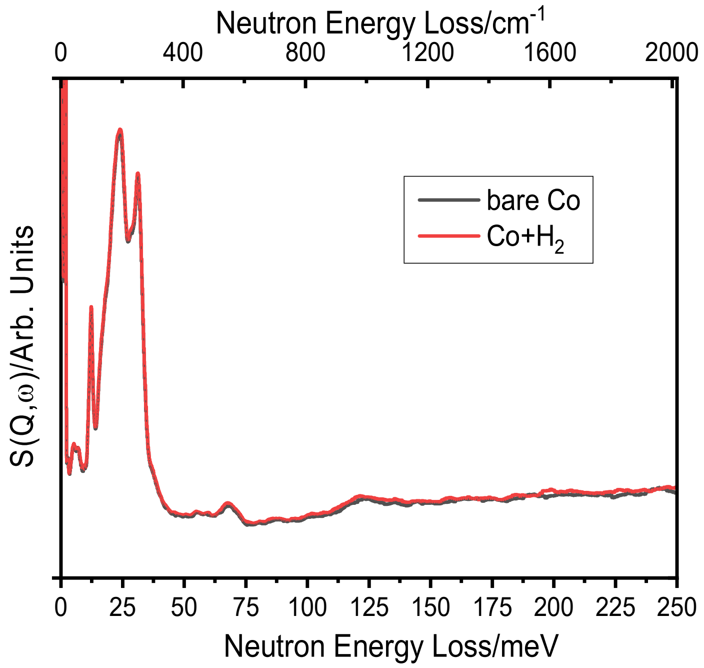

Supplementary Figure 10. Comparison of the experimental INS spectra for bare Co catalyst and the hydrogenated Co catalyst. The features below 300 cm^{-1} are contributed by the catalysis cell.

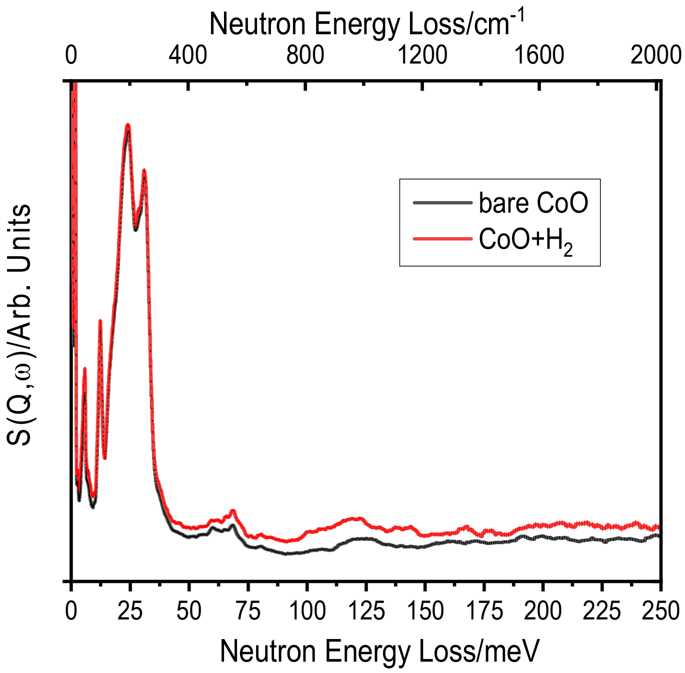

Supplementary Figure 11. Comparison of the experimental INS spectra for bare CoO catalyst and the hydrogenated CoO catalyst. The features below 300 cm^{-1} are contributed by the catalysis cell.

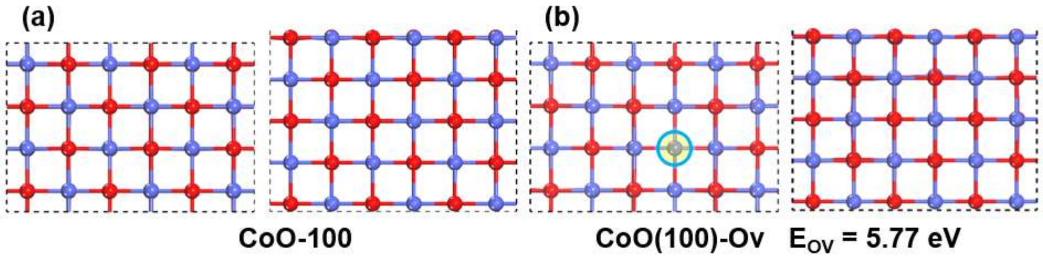

Supplementary Figure 12. Calculated structures. (**a**) CoO(100), (**b**) CoO(100)-O_V surfaces (Left: top view; Right: side view). O_V represents the missing oxygen atoms. Red: O, blue: Co; green circle represents the missing oxygens. These notations are used throughout the paper.

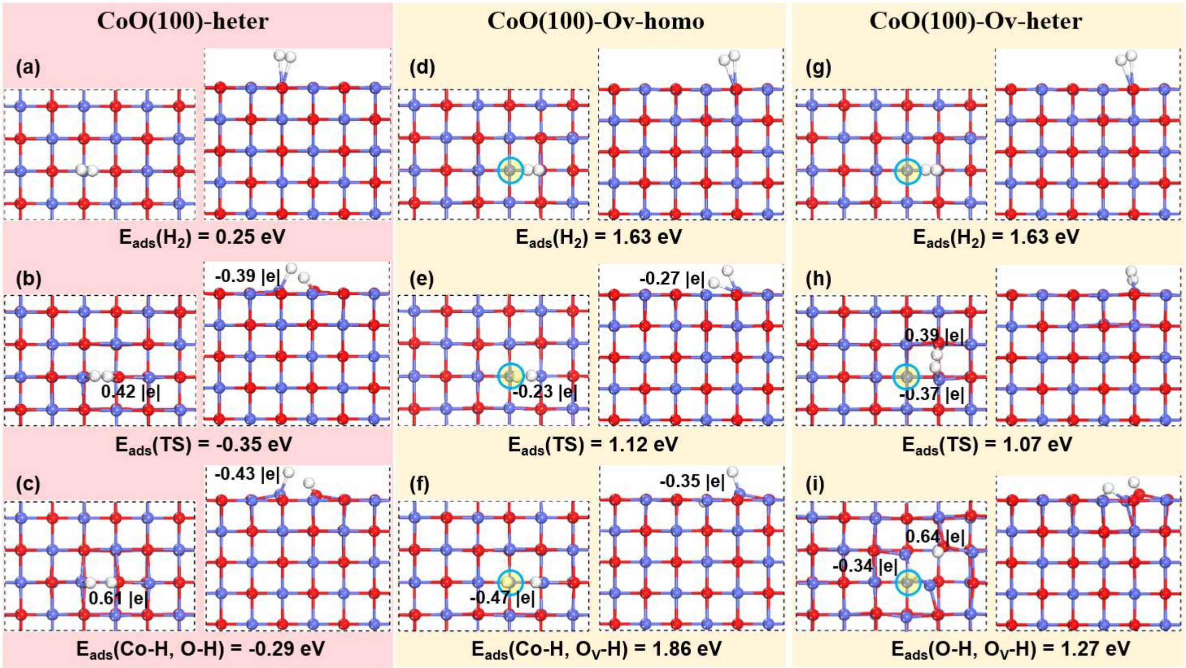

Supplementary Figure 13. **Calculated key structures (left: top view; right: side view) of H_2 adsorption and dissociation.** (**a**)–(**c**) at CoO(100) surface, which were marked with red background; (**d**)–(**i**) at CoO(100)-O_V surface, which were marked with yellow background. The calculated Bader charges of H species are also shown in (b-c, e-f, h-i).

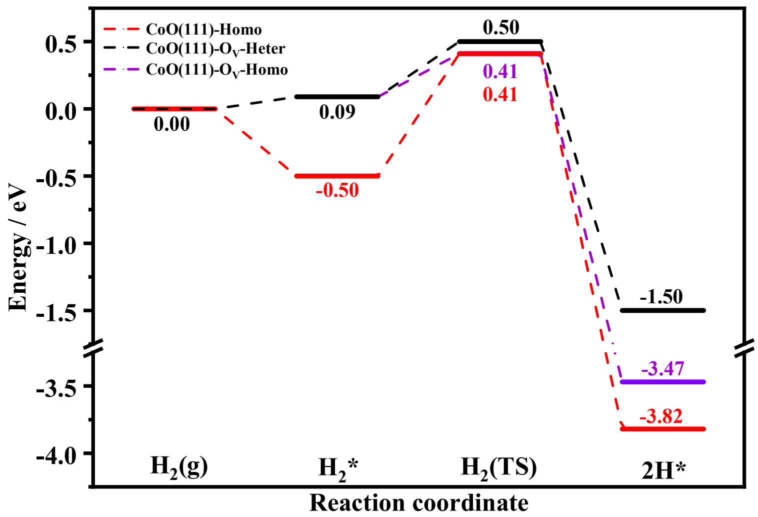

Supplementary Figure 14. **Calculated energy profiles of adsorption and dissociation of H_2 on the CoO(111) and CoO(111)-Ov surfaces.** H_2(g): gas-phase H_2; H_2*: adsorbed H_2 on surface; H_2(TS): the adsorbed H_2 on surface dissociates to two adsorbed H on surface; 2H*: the co-adsorption of two H on surface.

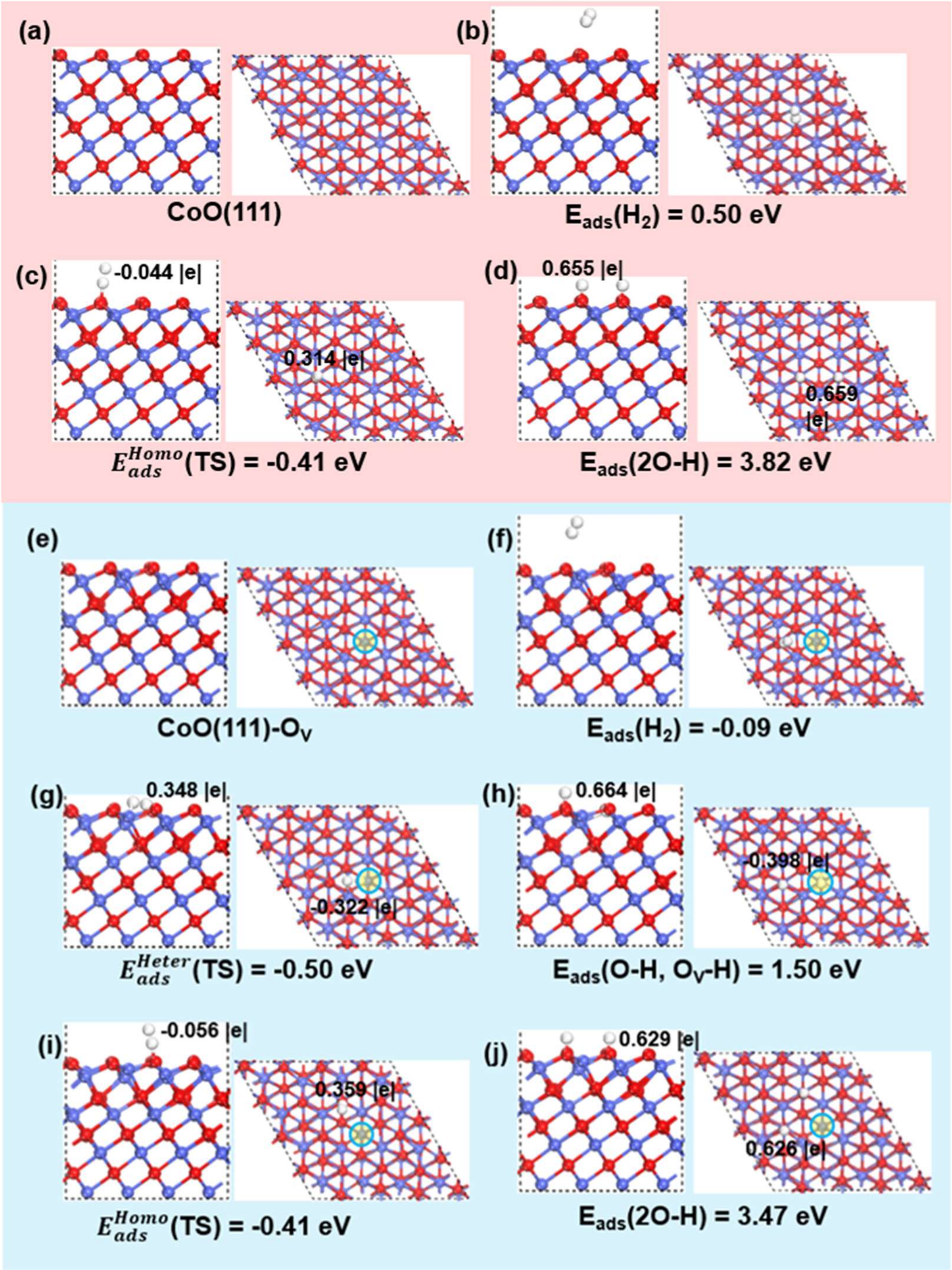

Supplementary Figure 15. Calculated key structures (left: top view; right: side view) of H_2 adsorption and dissociation. (**a**)–(**d**) at CoO(111) surface, which were marked with red background; (**e**)–(**j**) at CoO(111)-O_V surface, which were marked with blue background. The calculated Bader charges of H species are also shown in **c-d** and **g-j**.

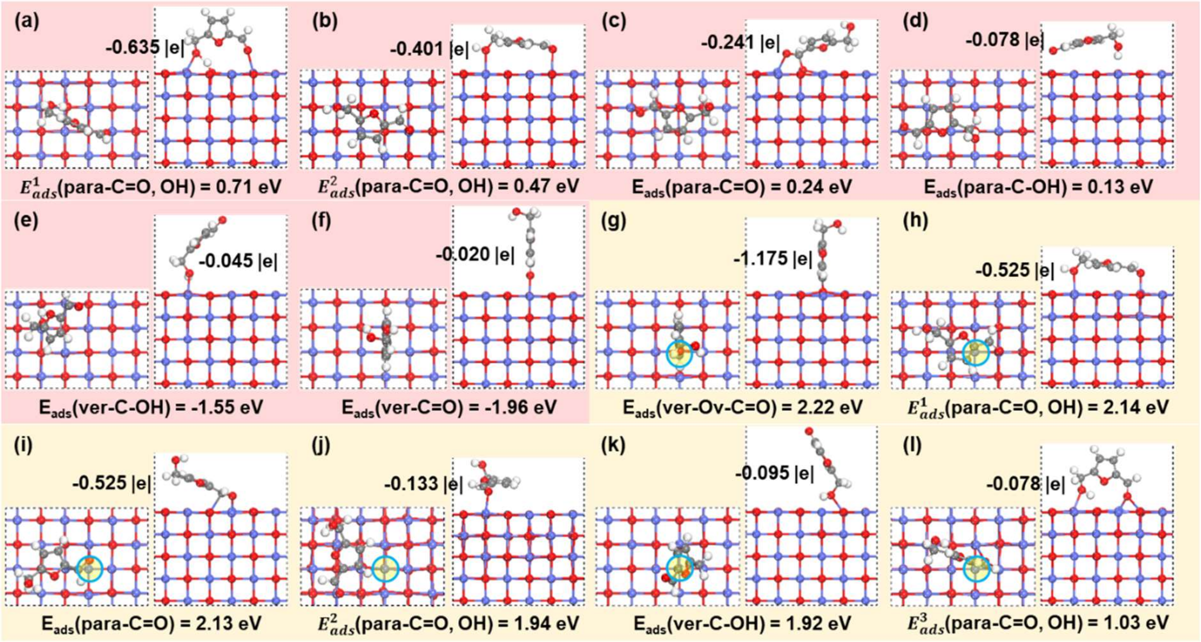

Supplementary Figure 16. **Calculated adsorption structures, energies, and Bader charges of HMF (left: top view, right: side view).** (**a**)–(**f**) at CoO(100) (red background); (**g**)–(**l**) at CoO(100)-O$_V$ (yellow background). E_{ads}(para-C=O, OH) represented the adsorption energy generated by the parallel adsorption of C=O and C-OH of HMF on catalyst surfaces. E_{ads}(para-C=O) represented the adsorption energy generated by the parallel adsorption of C=O of HMF on catalyst surfaces. E_{ads}(para-C-OH) represented the adsorption energy generated by the parallel adsorption of C-OH of HMF on catalyst surfaces. E_{ads}(ver-C-OH) represented the adsorption energy generated by the vertical adsorption of C-OH of HMF on catalyst surfaces. E_{ads}(ver-C=O) represented the adsorption energy generated by the vertical adsorption of C=O of HMF on catalyst surfaces. E_{ads}(ver-Ov-C=O) represented the adsorption energy generated by the vertical adsorption of C=O from HMF onto the oxygen vacancies on the CoO(100)-O$_V$ surface.

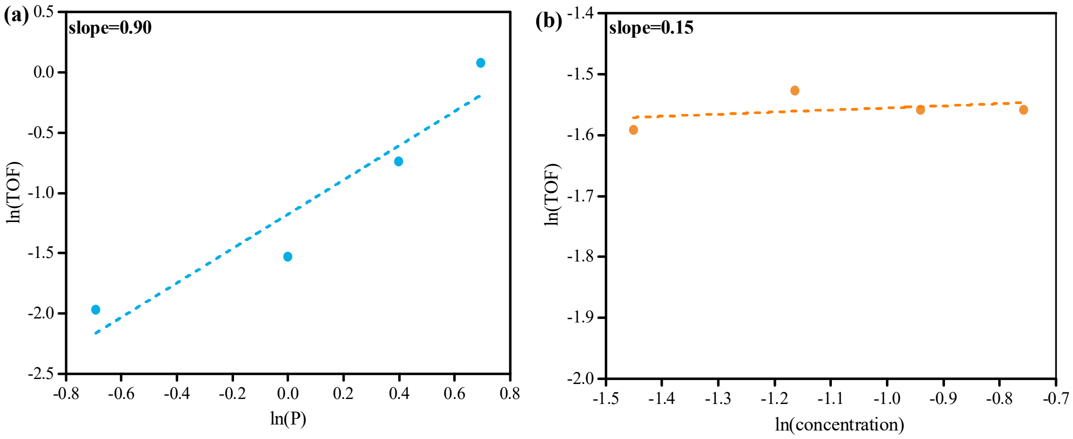

Supplementary Figure 17. Reaction kinetics for the HDO of 2,5-Furandimethanol using Co_3O_4-250. Reaction orders towards (**a**) H_2 and (**b**) substrate for the 2,5-furandimethanol HDO. Reaction conditions: 130 °C, 10 mg catalyst, 5.0 mL THF, (a) 0.5-2 MPa H_2, (b) 0.15-0.3 g 2,5-Furandimethanol, 1h reaction time.

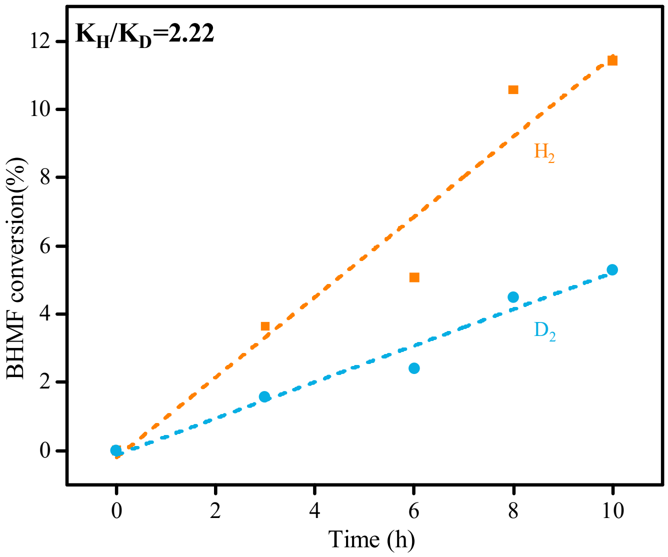

Supplementary Figure 18. Primary kinetic isotope effect observed for the HDO of 2,5-Furandimethanol. Reaction condition: BHMF, 200 mg; catalyst (Co_3O_4-400), 20 mg; THF, 5 ml; temperature, 130 °C; H_2/D_2, 1 MPa.

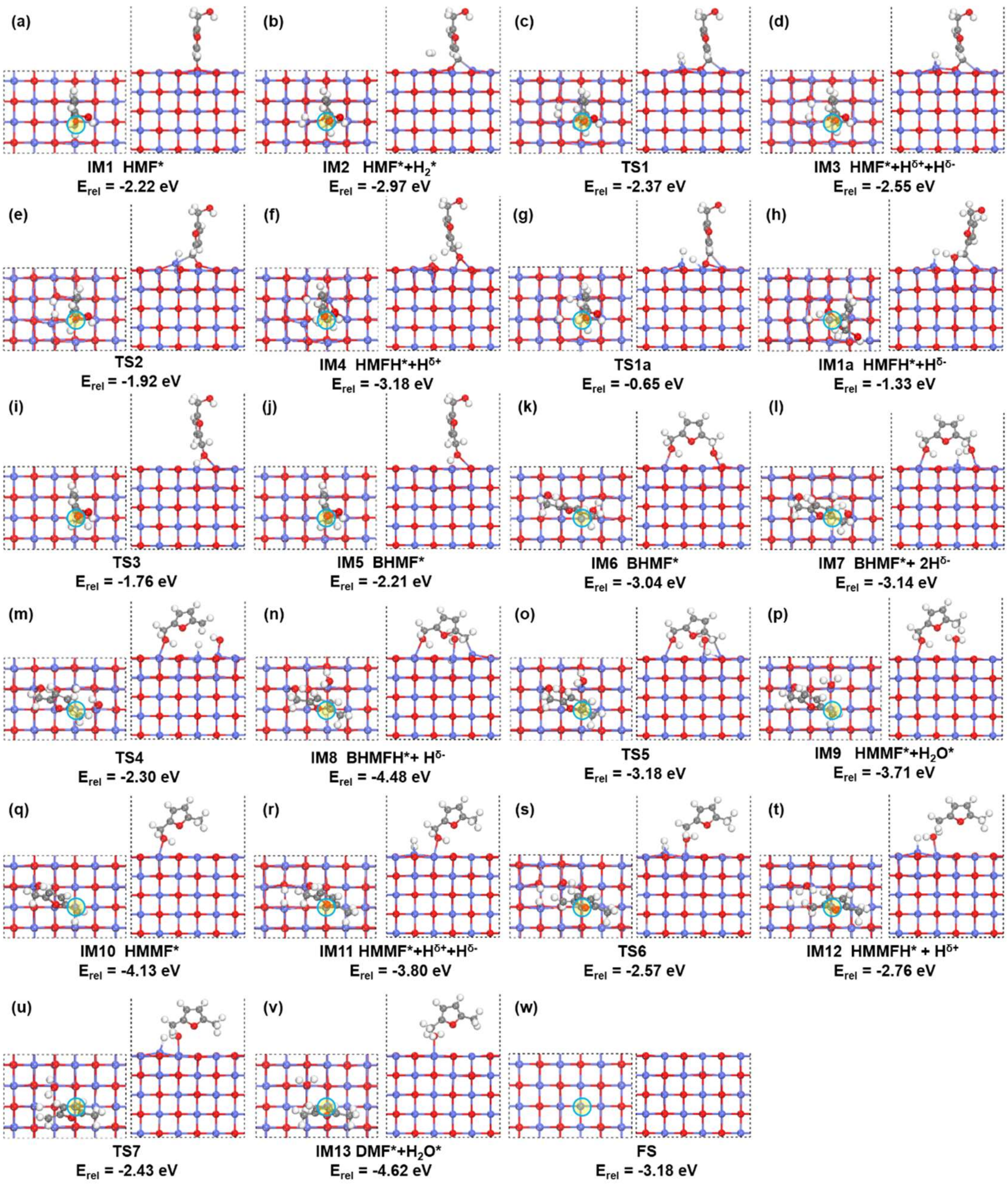

Supplementary Figure 19. Calculated structures and relative energies of the key species during HMF hydrogenolysis reaction on the CoO(100)-O$_V$ surface. (**a**) The adsorption structures of HMF; (**b-d**) H_2 adsorption and dissociation; (**d-j**) hydrogenation reaction of HMF to BHMF, and (**g-h**) the reaction route of $H^{\delta+}$ first attack; (**j-k**) the structure flip of BHMF; (**k-q**) hydrogenolysis reaction of BHMF to HMMF; (**r-v**) hydrogenolysis reaction of HMMF to DMF; (**w**) the finally state (CoO(100)-O$_V$ surface). The relative energies compared with the initial state (at E=0 eV). HMFH*, BHMFH* and HMMFH* represented the intermediate state, which was formed when the first hydrogen was added to the corresponding substrate (HMF*, BHMF* and HMMF*).

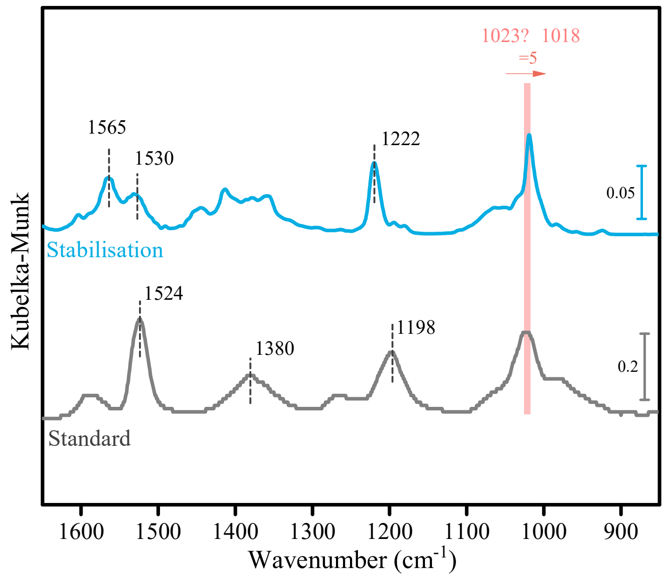

Supplementary Figure 20. DRIFTS spectra of 5-methyl-2-furanmethanol (HMMF) adsorbed on Co_3O_4-250 at 30°C followed by Ar flushing.

To analyze the interaction of substrate-to-catalyst and funan-ring-to-catalyst, HMMF-adsorption-IR analysis was carried out and the results are shown in Supplementary Figure 20. There is a red shift of the signal at 1023 cm^{-1} for C-O stretching in the alkoxy functional group[1,2]. This phenomenon indicated that Co_3O_4-250 catalysts has an activating effect on C-O bonds. In comparison, the characteristic peaks at 1500-1650 cm^{-1} for C=C stretching and 1198 cm^{-1} for C-O-C stretching, which are assigned to furan skeleton modes, show blue shifts. It suggests that the stretching vibration of C=C and C-O-C becomes stronger, which may be attributed to the activation of C-O bond by the alkoxy functional group[3].

Supplementary Tables

Supplementary Table 1. The evaluation of impact of solvents on the HMF hydrogenolysis reaction *

Solvent	Conv. (%)	Yield (%)				Mass balance (%)
		BHMF	HMMF	DMF	Others#	
THF	> 99	0.0	4.3	89.2	0.0	94.5
Ethanol	> 99	0.0	10.1	58.0	22.8	91.9
Isopropanol	> 99	0.2	13.8	63.5	16.4	94.9

* Reaction conditions: HMF: 0.15 g, Co_3O_4-250: 0.03 g, solvent: 5 mL, H_2: 1.0 MPa, 130 °C, 2 h.

# Others mainly include 5-methyl furfural (5-MF), ring hydrogenation by-products (MTHFA; DMTHF) and 2-hexanol.

The HMF conversion all reached 99% in 2h in these two solvents, comparable with that in THF, but the yield of DMF was lower, with HMMF as the intermediate and 5-methyl furfural(5-MF), ring hydrogenation by-products (MTHFA; DMTHF) and 2-hexanol as the by-products. The existence of HMMF may be due to the co-adsorption of hydroxyl groups in alcohols with that in intermediates on catalyst surface, and thus slow down the hydrogenolysis of intermediate to DMF (Supplementary Figure 5). The existence of byproducts may be attributed to the contribution of alcohols, which are knows as good hydrogen sources. Thus, these results indicate that the solvent can influence the catalytic performance and THF shows the best performance.

Supplementary Table 2. Comparison of a previously reported catalysts and Co_3O_4-250 for the HDO of HMF to DMF

	H_2 Pressure /MPa	Temp. /°C	t /h	5-HMF Conv. /%	Sel. to DMF /%	Yield of DMF /%	Productivity /mmol DMF·g^{-1}· h^{-1}	References
Ru-doped hydrotalcite	1	220	4	100	58.0	58.0	2.90	[4]
Ru/Co_3O_4	0.7	130	24	> 99	93.4	93.4	0.76	[5]
RuCo/CoO_x	0.5	200	2	100	96.5	96.5	19.13	[6]
Ru/CoFe-LDO	1	180	6	100	98.2	98.2	3.25	[7]
Pt_1/Co	1	180	2	100	92.9	92.9	2.93	[8]
Pt/rGO	3	120	2	100	73.2	73.2	9.15	[9]
PtCo@HCS	1	180	2	100	98	98.0	19.60	[10]
Pt-OMD1	3	200	6	99	62.9	62.3	4.15	[11]
Pd-OMD1	3	200	6	86.7	89.6	77.7	5.18	[11]
Pd/C/Zn	0.8	150	8	> 99	85	85.0	8.34	[12]
Raney Ni	1.5	180	15	100	88.5	88.5	1.40	[13]
Ni/C	4.5	180	2	100	75	75.0	6.00	[14]
Ni-OMD3	3	200	6	> 99.9	98.7	98.7	6.51	[11]
Ni/$LaFeO_3$	5	230	6	> 99	98.3	98.3	1.62	[15]
Raney Co	1.5	180	15	94.3	78.5	74.0	1.17	[13]
Ni-Co oxides	1	130	24	> 99	76.0	76.0	0.62	[16]
Ni-Co/C	1	130	24	99	95.0	94.0	0.39	[17]
2%Ni-20%Co/C	1.5	210	24	99	61.0	60.4	0.25	[18]
Co@C	5	180	8	100	91.9	91.9	11.39	[19]
Ag-Co@C(Ag:Co=1:3)	5	180	8	100	96.2	96.2	11.92	[19]
Zn-Co@C(Zn:Co=1:3)	5	180	8	100	91.5	91.5	11.34	[19]
Co-CoO_x	1	170	12	100	83.3	83.3	2.75	[20]
Co/Mix-ZrO_2	1	130	2	> 99	90.7	90.7	7.12	[21]
Raney Cu	1.5	180	15	25.1	42.0	10.5	0.17	[13]

Cu-Co/Al_2O_3(Cu/Co = 1)	3	200	8	99.9	68.4	68.4	0.43	[22]
CuZn	2	200	6	100	80.8	80.8	5.34	[23]
Cu-Ni/Al_2O_3(Cu/Ni = 1)	3	200	6	99	53	52.5	0.28	[24]
CuNi/TiO_2	2.5	200	8	100	84.3	84.3	1.39	[25]
Cu/Fe_2O_3-Al_2O_3	2	150	10	100	93.2	93.2	0.31	[26]
Co_3O_4-250	1	100	6	> 99.9	54.9	54.9	3.63	this work [a]
Co_3O_4-250	1	130	2	> 99.9	89.2	89.2	17.58	this work [a]
Co_3O_4-250	1	130	3	> 99.9	92.4	92.4	12.21	this work [a]
Co_3O_4-250	1	150	2	> 99.9	73.4	73.4	29.20	this work [b]
Co_3O_4-250	1	180	1	> 99.9	53.1	53.1	42.03	this work [b]

[a]reaction condition: HMF, 150 mg; catalyst (Co_3O_4-250), 30 mg.

[b]reaction condition: HMF, 300 mg; catalyst (Co_3O_4-250), 30 mg.

Supplementary Table 3. Calculated H-H bond distances in the process of H_2 dissociation and vibration frequencies of related transition states (TS) on CoO(100) surface and CoO(100)-O_V surface

	IS(H-H) / Å	TS(H-H) / Å	FS(H-H) / Å	Frequency / cm^{-1}
CoO(100)	0.76	1.08	1.59	-674.33
CoO(100)-O_V-heter	0.77	1.27	2.84	-1071.25
CoO(100)-O_V-homo	0.77	1.03	2.37	-851.02

In order to prove the calculation results reasonable or not, the H-H bond distances in the procedure of H_2 activation (including H_2 adsorption states, transition states as well as final states), and imaginary vibration frequencies of related transition states (TS) on the two models were calculated. The H-H bond distance was extended during the activation of hydrogen and the vibrational frequencies were all below -200 cm^{-1}, which confirms the calculation were reliable.

Supplementary Table 4. Conversion of lignin β-O-4 model compound over Co_3O_4 catalyst at different reduction temperature

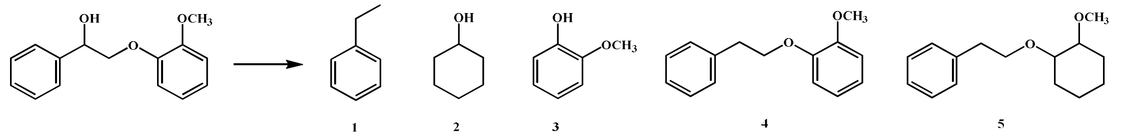

Catalyst	Yield (%)					Conv. (%)
	**1**	**2**	**3**	**4**	**5**	
Co_3O_4	32.1	1.7	30.5	64.3	--	100
Co_3O_4-200	39.8	27.6	11.0	52.2	--	100
Co_3O_4-250	78.9	77.0	--	13.0	1.2	100
Co_3O_4-300	5.2	0.6	3.0	88.2	0.2	100
Co_3O_4-400	4.0	--	3.1	83.0	--	92.6

Reaction conditions: substrate (200 mg), catalyst (100 mg), dioxane (5 mL), H_2 pressure (0.5 Mpa), temperature (180 °C), 4 h.

Supplementary Table 5. The evaluation of impact of solvents on the hydrogenolysis reaction of β-O-4 model compound*

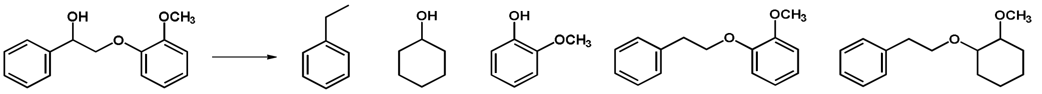

1 2 3 4 5

Solvent	Conv. (%)	Yield (%)					Mass balance (%)
		1	2	3	4	5	
1,4-Dioxane	100	78.9	77.0	--	13.0	1.2	93.1/91.2
THF	100	66.6	63.3	4.9	22.7	--	89.3/90.9
Ethanol	94.5	65.4	--	59.8	32.9	--	103.8/98.2

*Reaction conditions: β-O-4 model compound: 0.2 g, Co_3O_4-250: 0.1 g, solvent: 5 mL, H_2: 0.5 MPa, 180 °C, 4 h.

The catalytic activities and product distributions for the hydrogenolysis of lignin β-O-4 model compound in different solvents have been carried out. It is found that among all solvents, 1,4-dioxane showed the highest catalytic performance and the yield of ethylbenzene and cyclohexanol reach 78.9% and 77.0%, respectively, with nearly full conversion. A few by-products are detected, such as 1-methoxy-2-phenethoxybenzene (13.0%). When using THF as the solvent, the yield of ethylbenzene and cyclohexanol was only 66.6% and 63.3%, respectively. Meanwhile, the yield of 1-methoxy-2-phenethoxybenzene was up to 22.7%, giving lower hydrogenolysis activity than that in 1,4-dioxane. When ethanol was used as solvent, the conversion of lignin β-O-4 model compound is 94.5% and the products are predominantly benzene ring retaining products (ethylbenzene and guaiacol), which is consistent with the literature[27,28] These results indicate that the hydrogenolysis performance of lignin β-O-4 model compound is influenced by solvents, and dioxane shows the best performance.

Supplementary References

1. Yang, H., Yan, R., Chen, H., Lee, D. H. & Zheng, C. Characteristics of hemicellulose, cellulose and lignin pyrolysis. *Fuel*. **86**, 1781-1788, (2007).
2. Hu, J., Zhao, M., Jiang, B., Wu, S. & Lu, P. Catalytic Transfer Hydrogenolysis of Native Lignin to Monomeric Phenols over a Ni–Pd Bimetallic Catalyst. *Energy & Fuels*. **34**, 9754-9762, (2020).
3. Dong, L., Xia, J., Guo, Y., Liu, X., Wang, H. & Wang, Y. Mechanisms of $C_{aromatic}$-C bonds cleavage in lignin over NbO_x-supported Ru catalyst. *J. Catal.* **394**, 94-103, (2021).
4. Nagpure, A. S., Venugopal, A. K., Lucas, N., Manikandan, M., Thirumalaiswamy, R. & Chilukuri, S. Renewable fuels from biomass-derived compounds: Ru-containing hydrotalcites as catalysts for conversion of HMF to 2,5-dimethylfuran, *Catal. Sci. Technol.* **5,** 1463-1472 (2015).
5. Zu, Y., Yang, P., Wang, J., Liu, X., Ren, J., Lu, G. & Wang, Y. Efficient production of the liquid fuel 2,5-dimethylfuran from 5-hydroxymethylfurfural over Ru/Co_3O_4 catalyst, *Appl. Catal. B: Environ.* **146,** 244-248 (2014).
6. Gao, Z., Fan, G., Liu, M., Yang, L. & Li, F. Dandelion-like cobalt oxide microsphere-supported RuCo bimetallic catalyst for highly efficient hydrogenolysis of 5-hydroxymethylfurfural, *Appl. Catal. B: Environ.* **237,** 649-659 (2018).
7. Li, Q., Man, P., Yuan, L., Zhang, P., Li, Y. & Ai, S. Ruthenium supported on CoFe layered double oxide for selective hydrogenation of 5-hydroxymethylfurfural, *Mol. Catal.* **431,** 32-38 (2017).
8. Gan, T., Liu, Y., He, Q., Zhang, H., He, X. & Ji, H. Facile synthesis of kilogram-scale Co-alloyed Pt single-atom catalysts via ball milling for hydrodeoxygenation of 5-hydroxymethylfurfural, *ACS Sustainable Chem. Eng.* **8,** 8692-8699 (2020).
9. Shi, J., Wang, Y., Yu, X., Du, W. & Hou, Z. Production of 2,5-dimethylfuran from 5-hydroxymethylfurfural over reduced graphene oxides supported Pt catalyst under mild conditions, *Fuel* **163,** 74-79 (2016).
10. Wang, G.-H., Hilgert, J., Richter, F. H., Wang, F., Bongard, H.-J., Spliethoff, B., Weidenthaler, C., & Schüth, F. Platinum–cobalt bimetallic nanoparticles in hollow carbon nanospheres for hydrogenolysis of 5-hydroxymethylfurfural, *Nat. Mater.* **13,** 293-300 (2014).
11. Goyal, R., Sarkar, B., Bag, A., Siddiqui, N., Dumbre, D., Lucas, N., Bhargava, S. K. & Bordoloi, A. Studies of synergy between metal–support interfaces and selective hydrogenation of HMF to DMF in water, *J. Catal.* **340,** 248-260 (2016).

12. Saha, B., Bohn, C. M. & Abu-Omar, M. M. Zinc-assisted hydrodeoxygenation of biomass-derived 5-hydroxymethylfurfural to 2,5-dimethylfuran, *ChemSusChem*, **7,** 3095-3101 (2014).
13. Kong, X., Zhu, Y., Zheng, H., Dong, F., Zhu, Y., & Li, Y.-W. Switchable synthesis of 2,5-dimethylfuran and 2,5-dihydroxymethyltetrahydrofuran from 5-hydroxymethylfurfural over Raney Ni catalyst, *RSC Adv.* **4,** 60467-60472 (2014).
14. Gyngazova, M. S., Negahdar, L., Blumenthal, L. C. & Palkovits, R. Experimental and kinetic analysis of the liquid phase hydrodeoxygenation of 5-hydroxymethylfurfural to 2,5-dimethylfuran over carbon-supported nickel catalysts, *Chem. Eng. Sci.* **173,** 455-464 (2017).
15. Chen, M.-Y., Chen, C.-B., Zada, B. & Fu, Y. Perovskite type oxide-supported Ni catalysts for the production of 2,5-dimethylfuran from biomass-derived 5-hydroxymethylfurfural, *Green Chem.* **18,** 3858-3866 (2016).
16. Yang, P., Cui, Q., Zu, Y., Liu, X., Lu, G. & Wang, Y. Catalytic production of 2,5-dimethylfuran from 5-hydroxymethylfurfural over Ni/Co_3O_4 catalyst, *Catal. Commun.* **66,** 55-59 (2015).
17. Yang, P. P., Xia, Q. N., Liu, X. H. & Wang, Y. Q. High-yield production of 2,5-dimethylfuran from 5-hydroxymethylfurfural over carbon supported Ni-Co bimetallic catalyst, *J. Energy Chem.* **25,** 1015-1020 (2016).
18. Yang, P. P., Xia, Q. N., Liu, X. H. & Wang, Y. Q. Catalytic transfer hydrogenation/hydrogenolysis of 5-hydroxymethylfurfural to 2,5-dimethylfuran over Ni-Co/C catalyst, *Fuel* **187,** 159-166 (2017).
19. Chen, B. B., Li, F., Huang, Z. & Yuan, G. Carbon-coated Cu-Co bimetallic nanoparticles as selective and recyclable catalysts for production of biofuel 2,5-dimethylfuran, *Appl. Catal. B: Environ.* **200,** 192-199 (2017).
20. Li, D., Liu, Q., Zhu, C., Wang, H., Cui, C., Wang, C. & Ma, L. Selective hydrogenolysis of 5-hydroxymethylfurfural to 2,5-dimethylfuran over Co_3O_4 catalyst by controlled reduction, *J. Energy Chem.* **30,** 34-41 (2019).
21. Xiao, T., Liu, X., Xu, G. & Zhang, Y. Phase tuning of ZrO_2 supported cobalt catalysts for hydrodeoxygenation of 5-hydroxymethylfurfural to 2,5-dimethylfuran under mild conditions, *Appl. Catal. B: Environ.* **295,** 120270 (2021).
22. Srivastava, S., Jadeja, G. C. & Parikh, J. Influence of supports for selective production of 2,5-dimethylfuran via bimetallic copper-cobalt catalyzed 5-hydroxymethylfurfural hydrogenolysis, *Chin. J. Catal.* **38,** 699-709 (2017).

23. Bottari, G., Kumalaputri, A. J., Krawczyk, K. K., Feringa, B. L., Heeres, H. J. & Barta, K. Copper-zinc alloy nanopowder: a robust precious-metal-free catalyst for the conversion of 5-hydroxymethylfurfural, *ChemSusChem* **8,** 1323-1327 (2015).

24. Srivastava, S., Jadeja, G. C. & Parikh, J. Synergism studies on alumina-supported copper-nickel catalysts towards furfural and 5-hydroxymethylfurfural hydrogenation, *J. Mol. Catal. A-Chem* **426,** 244-256 (2017).

25. Seemala, B., Cai, C. M., Wyman, C. E. & Christopher, P. Support induced control of surface composition in Cu–Ni/TiO_2 catalysts enables high yield Co-conversion of HMF and furfural to methylated furans, *ACS Catal.* **7,** 4070-4082 (2017).

26. Esteves, L. M., Brijaldo, M. H., Oliveira, E. G., Martinez, J. J., Rojas, H., Caytuero, A., Passos, & F. B. Effect of support on selective 5-hydroxymethylfurfural hydrogenation towards 2,5-dimethylfuran over copper catalysts, *Fuel* **270,** 117524 (2020).

27. Lin, F. *et al.* Identification of the cleavage mechanisms and hydrogenation activity of the β-O-4 linkage in a lignin model compound over Ni-CeO_2/H-ZSM-5. *Appl. Catal. A: Gen.* **598**, 117552, (2020).

28. Chen, C., Liu, P., Xia, H., Zhou, M. & Jiang, J. Catalytic transfer hydrogenation of 4‐O‐5 models in lignin‐derived compounds to cycloalkanes over Ni‐based catalysts. *J. Chin. Chem. Soc*. **68**, 582-591, (2020).
